# Autoimmune Gastritis in the Pediatric Age: An Underestimated Condition Report of Two Cases and Review

**DOI:** 10.3389/fped.2018.00123

**Published:** 2018-05-01

**Authors:** Chiara Saglietti, Amedeo Sciarra, Karim Abdelrahman, Vanessa Schneider, Arti Karpate, Andreas Nydegger, Christine Sempoux

**Affiliations:** ^1^Institute of Pathology, Lausanne University Hospital, Lausanne, Switzerland; ^2^Gastroenterology and Hepatology Department, Lausanne University Hospital, Lausanne, Switzerland; ^3^Pediatric Gastroenterology Unit, Department of Pediatrics, Lausanne University Hospital, Lausanne, Switzerland

**Keywords:** autoimmune gastritis, gastric atrophy, children, intestinal metaplasia, ECL cell hyperplasia, iron-deficiency anemia

## Abstract

**Background:** Diagnosis of pediatric autoimmune gastritis (AIG) in children is important due to poor outcome and risk of malignancy. This condition is often underestimated in the clinico-pathologic diagnostic work-up, leading to delayed time-to-diagnosis. To increase the awareness of this condition in the pediatric population, we present two cases encountered at our institution, discuss their clinical, biological, and histological presentations in relation with evidence from the literature, and propose an algorithm for diagnosis and follow-up of AIG in children.

**Case presentation:** Two patients (12 and 17 years old) presented with iron deficiency anemia and negative family history for autoimmune disorders. In both cases, the final diagnosis of autoimmune gastritis was delayed until pathological examination of endoscopic gastric biopsies showed atrophy of oxyntic glands. *Helicobacter pylori* search was negative. Follow up biopsies revealed persistent disease. Literature review on this condition shows unclear etiology and poor long term outcome in some patients because of increased risk of malignancy.

**Conclusions:** AIG should be considered in the differential diagnosis of iron deficiency anemia in the pediatric population.Standardized clinico-pathologic work-up is mandatory. Endoscopic follow-up should be performed due to the risk of malignancy.

## Background

Autoimmune gastritis (AIG) is a chronic progressive inflammatory condition that is characterized by destruction of oxyntic glands and their replacement by atrophic and metaplastic mucosa, typically accompanied by lymphoplasmacytic infiltration of the lamina propria. The decrease or disappearance of parietal cells results in hypochlorhydria or achlorhydria and loss of intrinsic factor, which interfere with intestinal absorption of iron and vitamin B_12_, respectively (1). Iron and vitamin B_12_ malabsorption is responsible for a spectrum of clinical signs and laboratory alterations, including iron-deficiency (IDA), and megaloblastic anemia. Moreover, increased gastric pH relieves somatostatin-mediated inhibition of antral gastrin-producing cells, and the ensuing hypergastrinemia induces proliferation of enterochromaffin-like (ECL) cells, which may give rise to neuroendocrine tumors (2–4).

Although it has traditionally been considered as a disease predominantly affecting elderly women of Northern European descent, recent clinical reports have described this condition in pediatric patients, especially in association with refractory iron-deficiency anemia and other autoimmune diseases (5–14).

## Case presentation

### Case 1

A 12-year-old girl with a history of paleness, epigastric pain and menorrhagia, and no previous personal or family history of autoimmune disease, was referred to our hospital to evaluate a microcytic hypochromic anemia (Hb = 10.6 g/dl; MCV = 64 fl; MCH = 19.4 pg; MCHC = 302 g/l; iron = 2.7 μmol/l; ferritin = 6 μg/l) unresponsive to oral iron treatment. Because of positive fecal occult blood test and elevated fecal calprotectin (705 μg/g), she underwent upper and lower endoscopy, to look for sources of gastrointestinal bleeding. Colonoscopy and histological examination of colonic biopsies taken during the procedure were unremarkable. Upper endoscopy imaging was also unremarkable, but the histological examination revealed chronic atrophic gastritis at both fundic and antral level.

**Figure 1:**
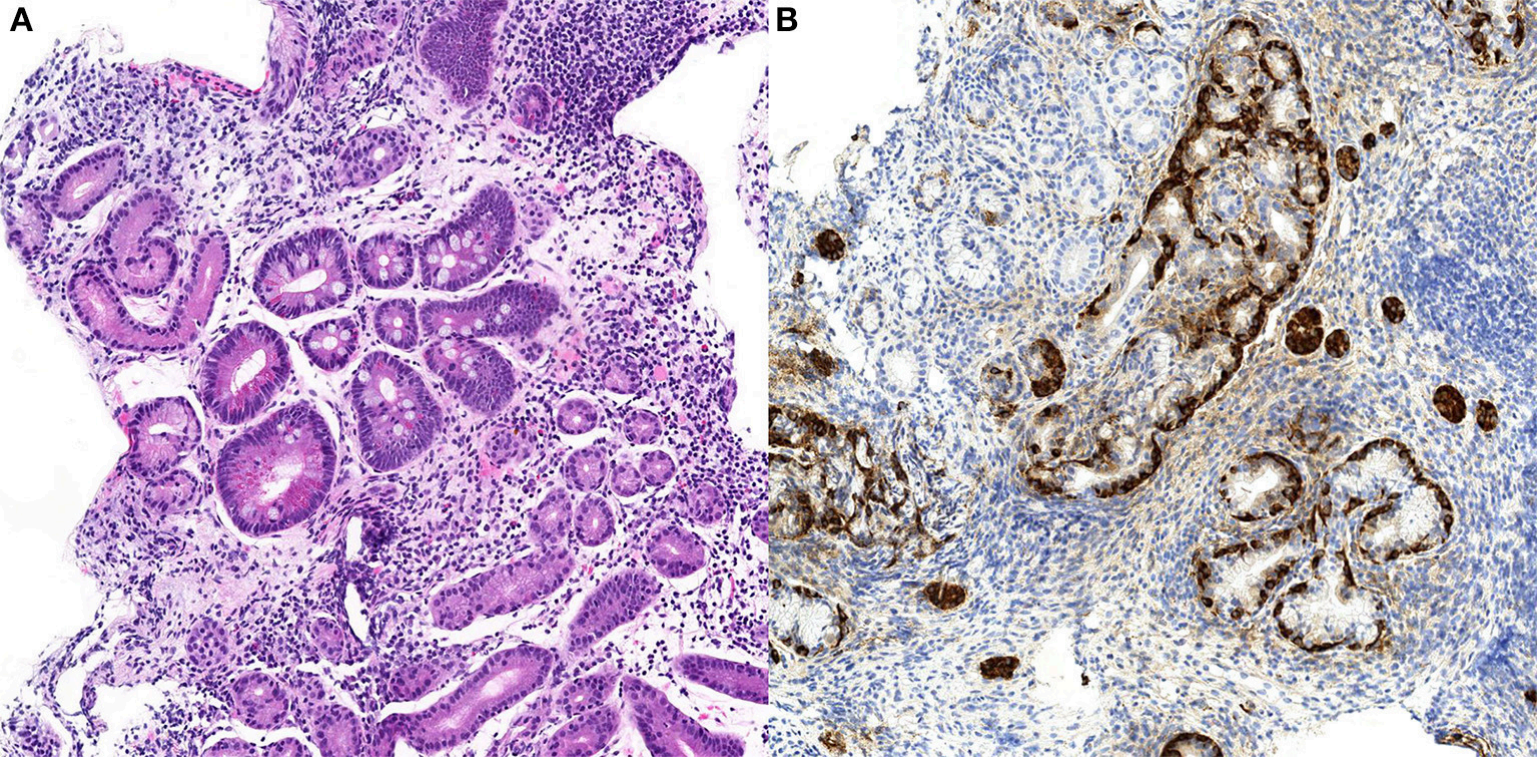
Case n.1 histological examination of gastric biopsies. At morphological analysis, fundic mucosa showed severe chronic gastritis, characterized by complete atrophy of the oxyntic glands and intestinal metaplasia, and lymphoplasmacytic predominant inflammatory infiltrate (**A**, H&E stain, 10x). Imunohistochemical staining for chromogranin A of the same biopsy demonstrated linear and micronodular hyperplasia of the ECL cells (**B**, chromogranin A stain, 20x).

Specifically, fundic biopsies showed a complete atrophy of the oxyntic glands and intestinal metaplasia, accompanied by a moderate inflammatory infiltrate composed of lymphocytes, plasma cells, eosinophils, and rare neutrophils. Linear and micronodular hyperplasia of ECL cells was demonstrated with immunohistochemical staining for chromogranin A. Antral biopsies exhibited a moderate chronic atrophic gastritis, without inflammatory activity and intestinal metaplasia. *Helicobacter pylori* search was negative after Giemsa-stained special coloration and immunohistochemical staining with anti-*Helicobacter pylori* specific antibody. A representative picture of these histological findings is shown in Figure 1.

Fecal *H. pylori* antigen test was positive, and the patient received a triple therapy with amoxicillin, clarithromycin for 14 days and esomeprazole for 1 month. Subsequent fecal antigen negative test confirmed *H. pylori* eradication. However, in a follow-up upper endoscopy performed 15 months later, histological examination proved unmodified. AIG was confirmed after further laboratory investigations revealing the presence of anti-parietal cell antibodies (1/1280 titer). Anti-intrinsic factor antibodies were negative and vitamin B_12_ levels were normal (164 pmol/l). Serum gastrin level was not assessed, as the patient was on esomeprazole, and urinary methylmalonic acid and homocysteine levels were not tested.

### Case 2

A 17-year-old girl with a history of menorrhagia, fatigue and dizziness, and no previous personal or family history of autoimmune disease, was referred to our hospital to evaluate an IDA (Hb = 6.7 g/dl; MCV = 56 fl; MCH = 16.1 pg; MCHC = 288 g/l; iron = 8.1 μmol/l) unresponsive to oral iron supplementation. Again, colonoscopy and upper endoscopy were unremarkable, but histological examination of gastric biopsies revealed chronic atrophic gastritis.

**Figure 2:**
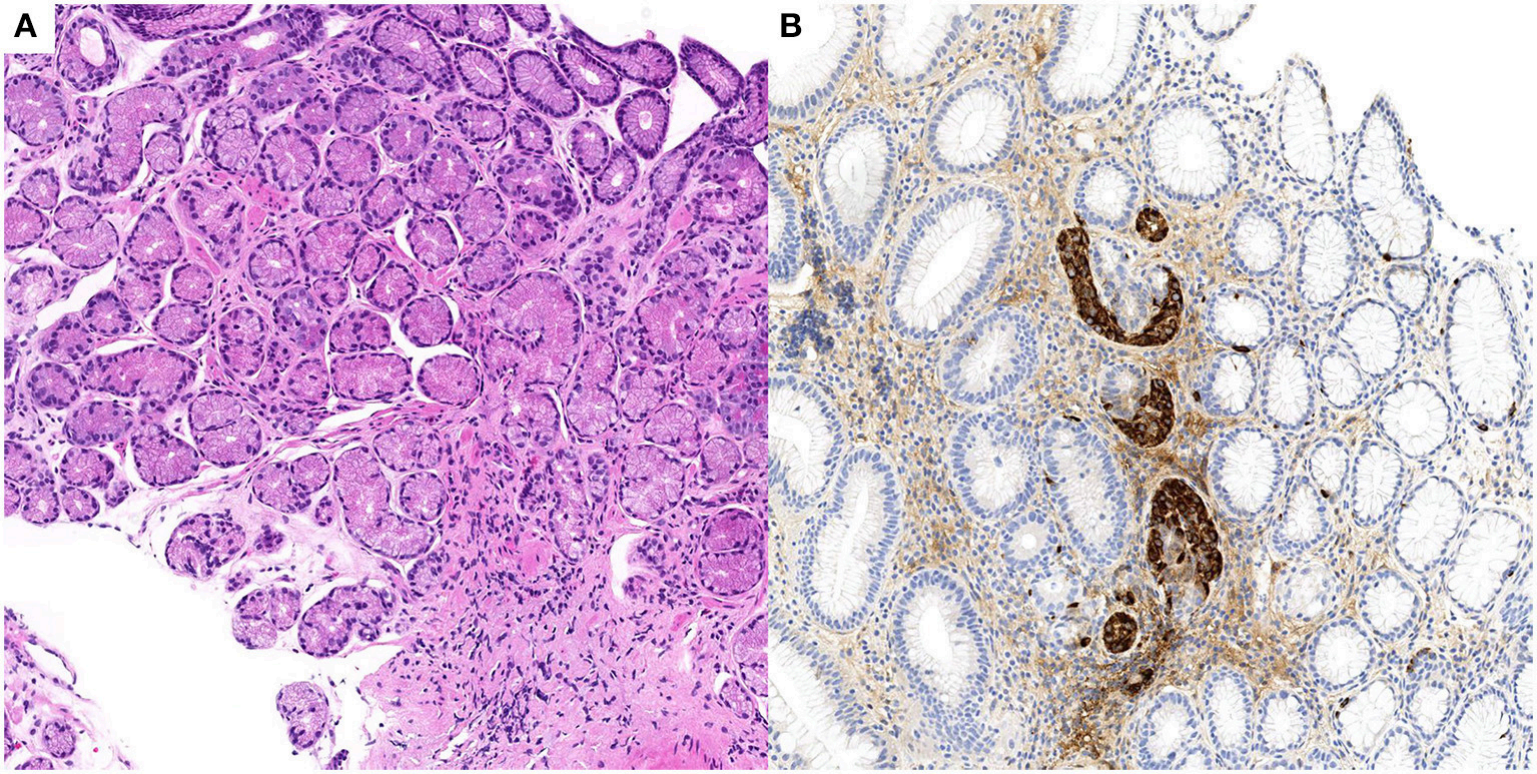
Case n.2 histological examination of gastric biopsies. At morphological analysis, fundic mucosa showed mild chronic gastritis, characterized by complete atrophy of the oxyntic glands and pseudopyloric metaplasia (**A**, H&E stain, 10x). Imunohistochemical staining for chromogranin A of the same biopsy demonstrated linear and micronodular ECL cell hyperplasia (**B**, chromogranin A stain, 10x).

Specifically, fundic biopsies showed complete atrophy of the oxyntic glands, with pseudo-pyloric metaplasia, accompanied by a mild inflammatory infiltrate composed of lymphocytes and plasma cells; micronodular hyperplasia of ECL cells was demonstrated with immunohistochemical staining for chromogranin A. *H. pylori* search was negative after Giemsa-stained special coloration and immunohistochemical staining with Anti-Helicobacter pylori specific antibody. Only mild chronic inflammation was observed in the antrum. A representative picture of these histological findings is shown in Figure 2.

After 7 months, a follow-up upper endoscopy histological examination demonstrated persisting fundic chronic atrophic gastritis with linear and micronodular hyperplasia of ECL. AIG was confirmed after further laboratory investigations revealing the presence of anti-parietal cell antibodies (1/2560 titer). Anti-intrinsic factor antibodies, as well as celiac disease or autoimmune thyroiditis specific serological tests, were negative. Vitamin B12 levels were normal (257 pmol/l). Urinary methylmalonic acid and homocysteine levels were not tested.

## Discussion and conclusions

In the cases reported here, AIG was a late and unexpected discovery in the diagnostic workup of a refractory anemia. This is in accordance with other previously published pediatric cases (Table 1), for which upper endoscopy was often delayed, in the context of refractory IDA, epigastric pain, or in the follow-up of other autoimmune diseases (6, 9, 11–16).

**Table 1:** Clinicopathological characteristics of patients with autoimmune gastritis (AIG) included in published case series and reports.

	**Sex**	**Age**	**Upper endoscopy indication**	**Endoscopic appearance**	**HP infection (histology)**	**Anemia**	**Iron deficiency**	**Vit B_12_ deficiency**	**Anti-PC Ab/ Anti-IF Ab**	**Hyper- gastrinemia**	**Metaplasia (follow-up)**	**ECL cell hyperplasia (follow-up)**	**Associated diseases**
Fröhlich-Reiterer pt #4	F	14.8	Anti-PC Ab > 100 U/ml	NA	No	Yes	Yes	No	Positive/NA	Yes	NA (NA)	No	Type 1 DM, Positive anti-thyroid autoantibodies
Fröhlich-Reiterer pt #5	F	18.5	Anti-PC Ab > 100 U/ml	NA	Yes	Yes	Yes	No	Positive/NA	No	NA (NA)	No	Type 1 DM, Positive anti-thyroid autoantibodies
Fröhlich-Reiterer pt #6	F	17.5	Anti-PC Ab > 100 U/ml	NA	Yes	Yes	Yes	No	Positive/NA	Yes	NA (NA)	No	Type 1 DM, Positive anti-thyroid autoantibodies
Fröhlich-Reiterer pt #7	F	14.8	Anti-PC Ab > 100 U/ml	NA	Yes	Yes	Yes	No	Positive/NA	No	NA (NA)	No	Type 1 DM, Positive anti-thyroid autoantibodies
Pogoriler pt #1	M	7	Chronic diarrhea, hypergastrinemia	Aspecific	No	No	No	No	Positive/Negative	Yes	No (Intestinal)	N (L, N)	AI hepatitis
Pogoriler pt #2	F	14	Irritable bowel syndrome	Aspecific	No	No	No	No	Positive/Positive	Yes	Pseudopyloric (Pseudopyloric)	L (L)	Type 1 DM
Pogoriler pt #3	M	7	Evaluation for CD	Aspecific	No	Yes	No	NA	NA/NA	NA	No (Intestinal) + AdenoCA	No (L)	CD, lymphocytic colitis, AI enterocolitis, DM, OP, AI pancytopenia
Pogoriler pt #4	M	13	Abdominal pain, weight loss	Aspecific	No	No	Yes	No	Negative/NA	NA	No (Intestinal)	No (No)	Evans syndrome, BOOP, CVID
Pogoriler pt #5	M	18	Vit B_12_ deficiency	Aspecific	No	No	No	Yes	NA/Positive	NA	No (No)	L, N (L, N)	DM, hypothyroidism
Pogoriler pt #6	F	17	Positive anti-PC Ab	Aspecific	No	Yes	Yes	NA	Positive/NA	NA	Pseudopyloric (NA)	No (NA)	DM, hypothyroidism
Pogoriler pt #7	F	0.7	Past cytomegalovirus gastritis	Aspecific	No	Yes	No	NA	Negative/NA	NA	NA (Squamous, mucinous)	NA (No)	Evans syndrome, T-cell immunodeficiency
Pogoriler pt #8	M	1.7	Gastritis/duodenitis follow-up	Aspecific	No	Yes	Yes	No	Negative/Negative	NA	Pseudopyloric (NA)	No (NA)	None
Pogoriler pt #9	F	17	IDA, suspected Crohn's disease	Aspecific	No	Yes	Yes	No	NA/NA	NA	No (NA)	No (NA)	Eating disorder
Pogoriler pt #10	F	14	Dyspepsia, anorexia	Aspecific	No	No	No	No	NA/NA	NA	Pseudopyloric (Pseudopyloric)	No (No)	None
Pogoriler pt #11	M	16	Chronic diarrhea, failure to thrive	Aspecific	No	No	Yes	No	Negative/Negative	NA	No (NA)	L, N (NA)	None
Pogoriler pt #12	F	14	Evaluation for CD	Aspecific	No	Yes	Yes	No	NA/NA	NA	No (NA)	No (NA)	None
Russell pt #1	F	15	Epigastric pain unresponsive to PPIs	Nodules …	No	No	NA	No	Negative/Negative	NA	Intestinal (Intestinal)	L, N (L, N)	None
Koca pt #1	F	15	Epigastric pain unresponsive to PPIs	Nodules …	Yes	No	NA	NA	Positive/NA	Yes	Intestinal (Intestinal)	L, N (L, N)	None
Kirsaclioglu pt #1	F	14	Recurrent IDA	Polyp …	NA	Yes	Yes	NA	Positive/NA	Yes	Instestinal (NA)	NET	None
Miguel pt #1	F	16	Refractory IDA	NA	Yes	Yes	Yes	NA	Positive/NA	Yes	No (NA)	NA (NA)	None
Miguel pt #2	F	16	Refractory IDA	NA	No	Yes	Yes	NA	Positive/NA	Yes	No (NA)	NA (NA)	None
Miguel pt #3	F	18	Refractory IDA	NA	Yes	Yes	Yes	NA	Positive/NA	Yes	No (NA)	NA (NA)	Positive anti-nuclear antibodies
Miguel pt #4	F	6	Refractory IDA	NA	No	Yes	Yes	NA	Positive/NA	Yes	No (NA)	NA (NA)	None
Miguel pt #5	F	4.7	Refractory IDA	NA	Yes	Yes	Yes	NA	Positive/NA	Yes	No (NA)	NA (NA)	None
Miguel pt #6	M	6	Refractory IDA	NA	No	Yes	Yes	NA	Positive/NA	Yes	No (No)	NA (NA)	None
Miguel pt #7	F	18	Refractory IDA	NA	Yes	Yes	Yes	NA	Positive/NA	Yes	Intestinal (NA)	NA (NA)	None
Miguel pt #8	M	13.8	Refractory IDA	NA	No	Yes	Yes	NA	Positive/NA	Yes	No (No)	NA (NA)	None
Gonçalves pt #1	M	14.75	Refractory IDA	Nodular duodenitis (2/5 patients) Fold softening (2/5 patients)	No	Yes	Yes	NA	Positive/NA	Yes	Intestinal (NA) [1/5 patients] Pseudopyloric (NA) [3/5 patients]	L (NA) [4/5 patients] N (NA) [3/5 patients]	None
Gonçalves pt #2	F	16	Refractory IDA		No	Yes	Yes	No	Positive/NA	Yes			None
Gonçalves pt #3	M	14.1	Refractory IDA		No	Yes	Yes	No	Positive/NA	Yes			Type 1 DM
Gonçalves pt #4	F	16.1	Refractory IDA		No	Yes	Yes	No	Positive/NA	No			None
Gonçalves pt #5	F	7.25	Refractory IDA		No	Yes	Yes	NA	Positive/NA	Yes			None
Katz pt	M	15	Weakness, appetite loss, IDA	Atrophic mucosa, polypoid mass	NA	Yes	Yes	Yes	NA/NA	NA	Intestinal + AdenoCA (NA)	NA (NA)	None
Greenwood pt #1	M	9	IDA	Erythematous gastric mucosa	No	Yes	Yes	No	Positive/Positive	Yes	No (NA)	No (No)	Addison's disease
Greenwood pt #2	F	8	Positive anti-PC Ab	NA	No	Yes	Yes	Yes	Positive/NA	No	Intestinal (NA)	No (No)	Primary AI hypothyroidism
Guilloteau pt	F	15	IDA	Pangastritis	NA	Yes	Yes	Yes	Positive/Positive	NA	Intestinal (NA)	NA (NA)	NA
Our patient #1	F	12	Refractory IDA, epigastric pain	Aspecific	No	Yes	Yes	NA	Positive/Negative	NA	Intestinal (Intestinal)	L, MN (MN)	Menorrhagia
Our patient #2	F	17	Refractory IDA	Unremarkable	No	Yes	Yes	No	Positive/Negative	NA	No (Pseudopyloric)	MN (L, MN)	Menorrhagia

*F, female; M, male; PC, parietal cell; IF, intrinsic factor; Ab, antibody; HP, H. pylori, Vit, vitamin; ECL, enterochromaffin-like; NA, not available; IDA, iron deficiency anemia; AdenoCA, adenocarcinoma; NET, neuroendocrine tumor; N, nodular; L, linear; MN, micronodular; DM, diabetes mellitus; CD, celiac disease; AI, autoimmune; OP, organizing pneumonia; BOOP, bronchiolitis obliterans organizing pneumonia; CVID, common variable immunodeficiency*.

When considering the whole of all pediatric patients reported in the literature, the average age at the diagnosis was 12.3 years, ranging from 8 months to 18 years. The most common presentations were refractory or recurrent IDA, followed by non-specific gastrointestinal symptoms. To further complicate the diagnosis, reported endoscopic findings were non-specific in most cases, and coexistence of positive anti-gastric parietal cells antibodies, vitamin B12 deficiency, and hypergastrinemia were present only in a minority of patients. Therefore, as in our cases, histologic examination was often the first analysis suggesting the possibility of AIG (9). It should be stressed that AIG is, by definition, a pathological process of the gastric body. Therefore, in the routine diagnostic workup of IDA, the clinician should always submit endoscopic biopsies from the gastric body and from the gastric antrum separately for pathological analysis. In the absence of fundic lesions, atrophia, and metaplastic changes occurring in the antrum are not a sign of AIG. Furthermore, fundic origin of the submitted biopsies has to be correctly identified by pathologists and special attention has to be paid not to misinterpret complete fundic atrophia as an antral specimen. For this specific purpose, a negative gastrin immunohistochemical staining can help to confirm the fundic origin of the samples. *H. pylori* infection, which is, to the best of our knowledge, the only differential diagnosis to consider should be attentively investigated, clinically, and pathologically.

As far as hematological alterations are concerned, pernicious anemia is not the only manifestation associated with AIG: by stratifying patients with AIG according to age, Hershko et al. suggested that IDA is the most frequent hematological presentation in young patients, and that there is a progressive increase in mean corpuscular volume and severity of vitamin B_12_ deficiency with advancing age at presentation (5). Young patients presenting with microcytic anemia are almost exclusively women, thus implying that menstrual blood loss may have a role in the development of iron deficiency, aggravated by the inability to increase food iron absorption due to hypochlorhydria. In effect, both of our patients had a history of menorrhagia, which could have contributed to their anemia (5).

The study of Hershko et al. has also shown a high prevalence of *H. pylori* positivity in young patients with AIG and microcytic anemia. Although the most common trigger of AIG in adults is *H. pylori* infection, with mechanisms of molecular mimicry hypothesized as initiating event of gastric autoimmunity on the basis of cross-reaction between *H. pylori* antigens and gastric H^+^,K^+^-ATPase, AIG appears to be a late complication of infection, as any ECL cell hyperplasia that may arise in that context (17). Therefore, the role of *H. pylori* infection in pathogenesis of AIG in pediatric population remains controversial. Although some authors have described remission of autoimmune atrophic gastritis after eradication of *H. pylori* infection in adults, we did not observe any improvement in gastric atrophy of our first patient after eradication therapy (18). The same observation was made by other authors who followed-up pediatric patients with *H. pylori* infection and AIG (9, 10). However, as *H. pylori* eradication is a simple procedure most of the times, regular search and treatment in case of positivity are recommended (8).

The long-term outcome of AIG in children has not been investigated yet. Our findings, in line with those of other authors, have shown no improvement in gastric atrophy, metaplasia, or ECL cell hyperplasia over time, consistently with known progression of AIG in adult patients (3, 4, 8, 10).

AIG is associated with intestinal-type gastric cancer and type I neuroendocrine tumors (3, 4). Consequently, it deserves particular attention as a preneoplastic condition in children, considering their long life expectancy. Different pathophysiological mechanisms are involved in the development of such gastric tumors: a pathological sequence of events progressing through inflammation, metaplasia and dysplasia ultimately results in intestinal-type gastric adenocarcinoma;(1) gastrin acts as a growth factor for ECL cells, and hypergastrinemia caused by achlorhydria induces ECL cell proliferations, which can develop into neuroendocrine tumor. In addition, ECL cells produce histamine, basic fibroblast growth factor and Reg protein, which are well-established to have trophic effects; therefore, it is reasonable to hypothesize that neuroendocrine cells play a role also in the pathogenesis of gastric cancer of the intestinal type (2). In published reports about neoplastic evolution of gastric atrophy in the pediatric population, one patient developed gastric adenocarcinoma at the age of 17 years, 8 years after initial diagnosis of AIG;(9) one patient developed a poorly differentiated gastric adenocarcinoma at 17 years, two and a half year after initial diagnosis;(11) a further patient presented at first upper endoscopy with a gastric neuroendocrine tumor in a background of autoimmune atrophic gastritis at the age of 14 (7).

**Figure 3:**
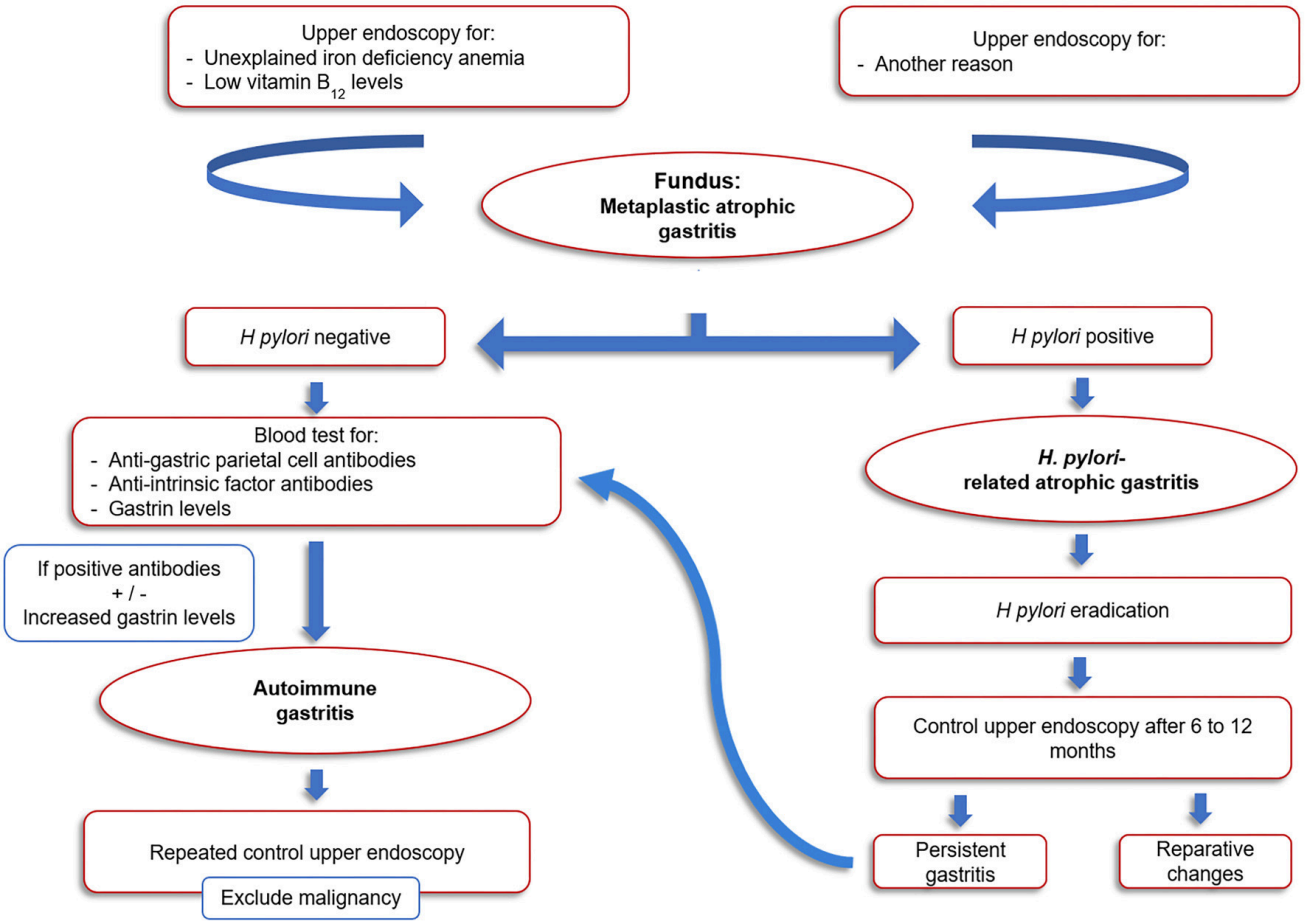
Algorithm suggested for the diagnosis and follow-up of pediatric autoimmune gastritis.

Since no curative therapy is available for AIG, treatment of this condition is focused on preventing vitamin B12 and iron deficiencies. Since dietary and oral iron supplements do not usually improve iron levels, alternative iron therapy approaches such as intravenous iron oral supplementation of ferrous glycine sulfate can be proposed (19). To date, the endoscopic follow up of AIG is still matter of debate and no specific guidelines for the pediatric population are available. For the adult population, the American Society for Gastrointestinal Endoscopy recommended in 2006 a single endoscopic evaluation for neoplastic lesions after diagnosis of AIG but no routine follow-up, while the guidelines from the European Society of Gastrointestinal Endoscopy recommends that once the diagnosis of AIG is established, follow-up endoscopies should be performed every 3 years to screen for the development of gastric malignancies (20, 21). Due to the longer life expectancy of the pediatric population and considering the relatively high risk of malignant transformation, surveillance protocols should be put in place for these patients. An algorithm for the diagnosis and follow-up of pediatric AIG is suggested in Figure 3.

In conclusion, physicians and pathologists should be aware of AIG existence also in the pediatric population. The histopathological examination can shorten the diagnostic delay, provided that adequate sampling of the stomach with body and antrum biopsies sent separately is performed. Poor outcome and risk of malignancy should be considered in the management strategy of these patients, taking into account their long life expectancy.

## Ethics statement

Samples were used in accordance with the Declaration of Helsinki. Written informed consent for publication of the case report was obtained from the patient or from the patient's parent/guardian, in the case of a minor.

## Author contributions

CS, AS, AN, and CSX: Study design, data collection, drafting. KA, VS, and AK: Data collection.

### Conflict of interest statement

The authors declare that the research was conducted in the absence of any commercial or financial relationships that could be construed as a potential conflict of interest.

## Abbreviations

AIG: autoimmune gastritis
IDA: iron-deficiency anemia
ECL: enterochromaffin-like
